# Predicting the effect of indirect cell kill in the treatment of multiple brain metastases via single‐isocenter/multitarget volumetric modulated arc therapy stereotactic radiosurgery

**DOI:** 10.1002/acm2.13400

**Published:** 2021-09-08

**Authors:** Allison N Palmiero, Denise Fabian, Marcus E Randall, William Clair, Damodar Pokhrel

**Affiliations:** ^1^ Medical Physics Graduate Program Department of Radiation Medicine University of Kentucky Lexington Kentucky USA

**Keywords:** direct/indirect cell kill, multiple brain metastases, setup uncertainties, SIMT‐VMAT SRS

## Abstract

**Purpose:**

Due to spatial uncertainty, patient setup errors are of major concern for radiosurgery of multiple brain metastases (m‐bm) when using single‐isocenter/multitarget (SIMT) volumetric modulated arc therapy (VMAT) techniques. However, recent clinical outcome studies show high rates of tumor local control for SIMT‐VMAT. In addition to direct cell kill (DCK), another possible explanation includes the effects of indirect cell kill (ICK) via devascularization for a single dose of 15 Gy or more and by inducing a radiation immune intratumor response. This study quantifies the role of indirect cell death in dosimetric errors as a function of spatial patient setup uncertainty for stereotactic treatments of multiple lesions.

**Material and Methods:**

Nine complex patients with 61 total tumors (2‐16 tumors/patient) were planned using SIMT‐VMAT with geometry similar to HyperArc with a 10MV‐FFF beam (2400 MU/min). Isocenter was placed at the geometric center of all tumors. Average gross tumor volume (GTV) and planning target volume (PTV) were 1.1 cc (0.02–11.5) and 1.9 cc (0.11–18.8) with an average distance to isocenter of 5.4 cm (2.2–8.9). The prescription was 20 Gy to each PTV. Plans were recalculated with induced clinically observable patient setup errors [±2 mm, ±2^o^] in all six directions. Boolean structures were generated to calculate the effect of DCK via 20 Gy isodose volume (IDV) and ICK via 15 Gy IDV minus the 20 Gy IDV. Contributions of each IDV to the PTV coverage were analyzed along with normal brain toxicity due to the patient setup uncertainty. Induced uncertainty and minimum dose covering the entire PTV were analyzed to determine the maximum tolerable patient setup errors to utilize the ICK effect for radiosurgery of m‐bm via SIMT‐VMAT.

**Results:**

Patient setup errors of 1.3 mm /1.3° in all six directions must be maintained to achieve PTV coverage of the 15 Gy IDV for ICK. Setup errors of ±2 mm/2° showed clinically unacceptable loss of PTV coverage of 29.4 ± 14.6% even accounting the ICK effect. However, no clinically significant effect on normal brain dosimetry was observed.

**Conclusions:**

Radiosurgery of m‐bm using SIMT‐VMAT treatments have shown positive clinical outcomes even with small residual patient setup errors. These clinical outcomes, while largely due to DCK, may also potentially be due to the ICK. Potential mechanisms, such as devascularization and/or radiation‐induced intratumor immune enhancement, should be explored to provide a better understanding of the radiobiological response of stereotactic radiosurgery of m‐bm using a SIMT‐VMAT plan.

## 1 INTRODUCTION

Due to fast treatment delivery, single‐isocenter/multitarget (SIMT) volumetric modulated arc therapy (VMAT) stereotactic radiosurgery (SRS) has become an increasingly popular treatment modality in the management of multiple brain metastases (m‐bm).^1^, ^2^, ^3^ Recently, this approach has been adopted and automated by Varian (Varian Medical Systems, Palo Alto, CA) in the Eclipse treatment planning system (TPS, version 15.6) as the HyperArc module, which has generated global clinical interest.^4^, ^5^, ^6^, ^7^, ^8^, ^9^ SIMT‐VMAT reduces treatment times while improving patient comfort and clinic workflow; however, there are concerns with patient setup uncertainty when treating multiple targets simultaneously. It has been previously demonstrated that clinically unacceptable dosimetric discrepancies due to rotational setup errors were present compared to treating each lesion individually.^10^, ^11^, ^12^, ^13^, ^14^, ^15^, ^16^, ^17^, ^18^, ^19^ The most recent simulation study demonstrated that there was a large loss of target(s) coverage (30% average, but up to 70% for small lesions) due to both rotational and translational setup errors while using SIMT‐VMAT SRS for m‐bm.^20^ This is a challenge in lining up all tumors correctly using a single daily cone‐beam CT scans, especially since skull‐based rigid alignment is required. In addition, soft tissue visibility is low inside the brain on low‐quality cone‐beam CT images. Targets may slightly move if there is intracranial edema. Nevertheless, a recent clinical study by Palmer et al. demonstrated positive clinical outcomes of SIMT treatments.^21^ They reviewed 173 patients treated with one to five brain lesions that underwent single‐isocenter SRS treatments. After an average of 12 months following up interval, very promising 1‐ and 2‐year tumor local control rates of 99 and 95% were observed. Other clinical studies have observed similar patient outcomes demonstrating SIMT‐VMAT SRS for m‐bm to be both safe and effective with high rates of tumor response.^22^, ^23^, ^24^, ^25^

However, the presence of positive clinical outcomes cannot be fully explained knowing the effects of patient positioning uncertainties in SIMT‐VMAT treatments. Biological modeling, specifically with the widely used linear‐quadratic (LQ) model could potentially overestimate the tumor control rate with SRS techniques. This is due to the LQ cell survival curve bends continuously downward (without limit) with increases in radiation dose because of the intrinsic quadratic component in the model.^26^, ^29^ This suggests that mechanisms in addition to tumor DNA double‐strand breaks and/or chromosomal aberrations must be involved. It is hypothesized that in addition to direct cell kill (DCK), the effect of indirect cell kills (ICK) could be playing a major role in SRS treatments. There are three types of indirect cell death to consider: strand breaks by free radicals, antitumor immunologic rejection, and devascularization.^27^ A majority of the literature suggests that cell death happened soon after irradiation, pointing toward devascularization as the mode of ICK.^26^, ^27^, ^28^, ^29^ For instance, Song et al. performed a study to connect the effects of radiobiological response on SBRT and SRS treatments.^29^ They concluded that irradiation of tumors with doses higher than 15 Gy per fraction causes major vascular damage accompanied by deterioration of intratumor microenvironment resulting in secondary tumor cell death. Other studies had similar findings.^30^, ^31^, ^32^, ^33^, ^34^ Tumor vasculature is disorganized with weak and fragile cellular walls consisting of thin tumor vessels with irregularly shaped endothelial cells in only a single layer with large gaps. These vessels are very susceptible to ionizing radiation, subjecting tumor vasculature to radiation damage when exposed to a single high‐dose (15 Gy or higher) results in the inverted “hockey‐stick” phenomena.^29^, ^32^, ^33^ With this theoretical phenomenon, the bend of the cell survival curve increases, where cell death is increasing at higher doses of radiation. When considering the effects of ICK, the local tumor control rates with the presence of dosimetric discrepancies due to patient setup errors in SIMT‐VMAT treatments have yet to be explored.

Therefore, to provide dosimetric support for the potential contribution of secondary cells death in the treatment of m‐bm with SIMT‐VMAT, a model has been created to define the relationship between spatial setup uncertainty and the delivered dose. Given the previous studies suggesting high levels of vascular damage at 15 Gy, the 15 Gy isodose volume (IDV) around the tumor was chosen as a threshold dose that best utilizes the effects of ICK in addition to DCK. As long as the target receives a minimum dose of 15 Gy or higher, vascular damage could theoretically influence indirect tumor cell death. This study attempts to characterize the patient setup errors that should be maintained in the treatment of m‐bm via SIMT‐VMAT to account for both effects of DCK and ICK. Although the previous studies have demonstrated the dosimetric effects of translational and rotational errors, they did not provide the effect of ICK in the treatment of m‐bm using SIMT‐VMAT plans.^10^, ^11^, ^12^, ^13^, ^14^, ^15^, ^16^, ^17^, ^18^, ^19^, ^20^ Therefore, the relationship between ICK and patient setup errors was used to define an uncertainty cut‐off value. It is clinically useful to know the allowable setup uncertainties for multilesion SRS treatments. This may provide limits on patient setup uncertainty that physicians could consider, providing them some clinical guidance in their SIMT‐VMAT treatments for treating m‐bm simultaneously.

## 2 MATERIALS AND METHODS

### 2.1 Patient information

Nine complex patients with 2–16 (61 total) brain metastases (all lung primary) were included in this study approved by our institutional review board. These patients were previously treated through single‐fraction SRS. High‐resolution double contrast MPRAGE MRI images (Siemens MAGNETOM, 1.5T MRI System, Ferndale, MI) were used for tumor and organs at risk (OAR) delineation and were coregistered to planning CT images in the Varian Eclipse TPS. The MPRAGE MRI images were 512 × 512 pixels with 1‐mm slice thickness and no gap in between slices. The target volumes were delineated by an experienced radiation oncologist on the MRI with the gross tumor volumes (GTVs) defined by the visible tumor. The planning target volumes (PTVs) were created using a uniform 1.0 mm margin around each GTV using departmental SRS protocol. The tumor characteristics are summarized in Table 1. The normal brain was considered all tissue with the GTVs included. Additionally, nearby OAR (hippocampi, brainstem, and optics apparatus) were contoured for dose reporting. Distance to isocenter was calculated as the 3D Euclidian distance from the isocenter and the lesion. The average distance to isocenter was 5.4 cm (range: 2.2 ‐ 8.9 cm) as shown in Table 1.

**TABLE 1:** Main tumor characteristics of the patients included in this study

Patient no.	No. of lesions	Avg. distance to isocenter (cm)	Total GTV (cc)	Total PTV (cc)	Adjacent OAR
I	2	2.2	2.2 ± 0.78	3.70 ± 1.10	Hippocampi
II	3	5.7	0.43 ± 0.78	0.93 ± 0.78	Hippocampi
III	4	6.5	3.90 ± 5.20	6.50 ± 8.30	Hippocampi, Optic apparatus
IV	5	8.9	3.30 ± 3.30	4.90 ± 4.30	Hippocampi
V	6	5.4	1.30 ± 0.72	2.10 ± 1.10	Hippocampi
VI	7	5.0	0.75 ± 0.81	1.40 ± 1.20	Brainstem, Hippocampi
VII	8	4.3	0.51 ± 0.58	1.00 ± 0.93	Brainstem, Hippocampi
VIII	10	5.5	0.39 ± 0.46	0.86 ± 0.46	Hippocampi
IX	16	5.4	0.43 ± 0.63	0.83 ± 1.00	Optic apparatus, Hippocampi, Brainstem

### 2.2 SIMT‐VMAT plans

SIMT‐VMAT SRS plans were generated in the Eclipse TPS for the TrueBeam LINAC (Varian Medical Systems, Palo Alto, CA) with a 10 MV flattening filter‐free (FFF) beam (2400 MU/min). A HyperArc style, fixed‐geometry was mimicked with three noncoplanar partial arcs and one full arc with couch positions at 0°, 45°, 315°, and 270°. The isocenter position was chosen at the approximate geometric center of all targets. Patient‐specific collimator angles were manually assigned to best minimize island blocking and dose spill outside of the target(s). The prescription was 20 Gy to each lesion to the 70–80% isodose line and optimized so that 95% of the target volume receives 100% of the prescription dose. The dose was calculated using Anisotropic Analytic Algorithm (AAA) (Eclipse, version 15.6) with the smallest calculation grid size of 1.25 mm. Ring structures to each target, jaw tracking, and normal tissue objective were used during inverse optimization for dose steering and to maintain dose fall‐off outside the target(s). Hippocampi were spared following RTOG‐0933 protocol's (equivalent dose to SRS) along with the optics apparatus and brainstem meeting QUANTEC guidelines.^35^, ^36^, ^37^

### 2.3 Simulated SIMT‐VMAT plans

Clinically observable patient setup uncertainties of ±0.5 mm/0.5°, ±1 mm/1°, and ±2 mm/2° in all six degrees‐of‐ freedom (6 DOF) were systematically simulated by using an in‐house registration method. Rotational errors were defined as the pitch (*y*‐*z* plane), roll (*x*‐*z* plane), and yaw (*x*‐*y* plane) relative to the isocenter position. After the SIMT‐VMAT plans were generated, the image set was duplicated and reregistered to the original MRI images. This registration was exported from the Eclipse TPS as a DICOM file and imported into a MATLAB script (Mathworks Inc., WA, USA). The script generated a matrix with rotational (Δ*α*, Δ*β*, Δ*γ*) and translational (Δ*x*, Δ*y*, Δ*z*) values and was applied to the reference frame. A new image registration DICOM file was generated and then imported back into the Eclipse TPS with a new transformation matrix applied. The original plan was then overlaid on to the new transformation and the dose was recalculated with the only difference being the isocenter shift.

### 2.4 Modeling direct versus ICK

This work attempts to model the effects of cell killing due to both direct and ICK methods. An assumption is made that for areas of the target receiving the prescription dose, 20 Gy or higher, tumor death is due to primarily direct cell killing methods, or DNA double strand breaks. Alternatively, for areas of the target receiving 15 Gy or higher, it is hypothesized that the tumor cell death is largely due to the ICK method via devascularization of the tumor and deteriorating the intratumor microenvironment. This threshold for ICK comes from the literature, as mentioned previously. Doses above 15 Gy could result in vasculature damage and, therefore, indirectly killing the tumor cells.^26^, ^27^, ^28^, ^29^, ^30^, ^31^, ^32^, ^33^ These assumptions are made to simplify the model, though, realistically, combinations of both direct and ICK are present.

**FIGURE 1:**
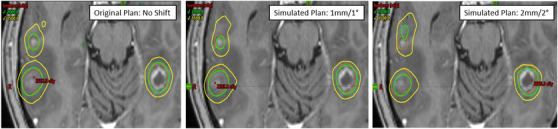
Example patient's SIMT‐VMAT plan with 16 lesions and a prescription of 20 Gy to each lesion. The left panel shows the original plan with no induced setup uncertainties, the middle panel shows a simulated plan with 1 mm/1° residual patient setup errors and the right panel shows another simulated plan with 2 mm/2° setup uncertainty in all 6 DOF. The orange contour is the PTV(s), the green isodose line is the prescription dose (20 Gy), and the yellow isodose line is 15 Gy. The simulation plans show decreasingly less coverage by the 20 Gy isodose line compared to the original plan, demonstrating the dosimetric effects of setup errors and the contribution of ICK

Both DCK and ICK methods were modeled using Boolean operators in the contouring module of the Eclipse TPS. For each PTV, the 20 and 15 Gy IDV were exported from the original SIMT‐VMAT plan and each of the simulated plan for the corresponding patient setup errors. Boolean operators were used to determine the overlap of the 20 Gy IDV and each PTV. This volume was denoted as the volume of the PTV receiving DCK. Another Boolean operator was used to find the overlap of the 15 Gy IDV and each PTV minus the 20 Gy IDV overlap with the target. This volume was signified as the volume of the tumor that was receiving primarily ICK effects. The concepts are further illustrated in Figure 1. This is an example of a patient with 16 lesions. The orange contour is the PTVs, the green isodose line is the prescription dose (20 Gy), and the yellow isodose line is the 15 Gy. For the original plan, the PTV is well covered by the prescription dose, therefore, should receive the greatest effects of DCK. However, the simulated plan with the setup errors of 1 mm and 1° shows a slight deviation of the 20 Gy isodose line, but the tumor is still covered entirely by the 15 Gy line (see Figure 1). This should result in a combination of both direct and ICK in this patient's treatment, which could result in a positive local tumor control rate. Furthermore, the simulated plan with 2 mm/2° setup errors has shown the significant loss of target coverage by the 20 Gy isodose line, but still displays a majority of the PTV coverage by the 15 Gy isodose line. With these large setup errors, the lesions will have a decreased target coverage, therefore, lower rate of tumor local control, even when considering the effects of ICK.

### 2.5 Data analysis

None of these simulated SRS patients were treated with the SIMT‐VMAT plans. This simulation study sought to find the maximum tolerable patient setup errors to fully utilize the effects of both direct and ICK to achieve acceptable local tumor control in the SIMT setting. Boolean structures of IDV were created iteratively until a dose was found to just fully cover the target. This process was repeated for the original SIMT‐VMAT plans and each of the corresponding simulated VMAT plan. Doses found to cover the target with 15 Gy IDV and above were deemed acceptable and assumed to generate a positive local tumor control rate via ICK. The roles of DCK versus ICK were also compared for each tumor. These were defined by creating Boolean structures for both the 15 and 20 Gy IDV as further described in the previous section. The volumes of these structures were taken and compared as a percentage of the PTV volume receiving that dose. These values were compared for the original SIMT‐VMAT plans and each of the corresponding simulated VMAT plans with the clinically realistic patient setup errors.

It is also of clinical interest to compare the effect that patient setup uncertainties have on normal brain dose and the role it could play in radionecrosis. For this reason, the normal tissue (brain) complication probability (NTCP) was modeled based on a study by Milano et al.^38^ This group pooled published reports of clinical data of radiation‐induced brain toxicity after receiving brain SRS treatments (single and multiple fractions). The data were fitted and a logistic model was used to create a usable NTCP function given by the following relation:

$$NTCP\;=\frac{1}{1+{\left(\frac{V_{x50}}{Vx}\right)}^{4\gamma_{50}}}$$

where Vx is considered the volume receiving greater than or equal to a dose of x Gy and $V_{x50}\;$is the volume corresponding to 50% risk of radionecrosis with $\gamma_{50}$ as a slope parameter. The values of $V_{x50}$ and $\gamma_{50}$ were taken from their NTCP model for brain metastases.^38^ In addition to changes in doses to normal brain and the maximum dose to OAR due to patient setup errors were also reported.

## 3 RESULTS

**FIGURE 2:**
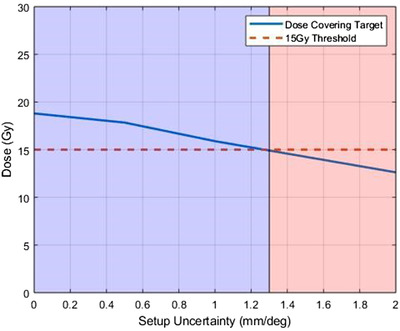
Illustration of the dose to target(s) for the original SIMT‐VMAT plans and the corresponding simulated VMAT plans with 0.5 mm/0.5°, 1 mm/1°, and 2 mm/2° residual patient setup errors. The blue line represents the dose that fully covers the target and the dotted red line represents the 15 Gy ICK threshold. The section of the plot covered in blue represents the target(s) coverage that is above the 15 Gy threshold, and the orange is below 15 Gy. Patient setup errors must be limited to those defined by the blue area

Figure 2 demonstrates the setup uncertainty limitations for the target (PTV) to be fully covered by at least 15 Gy or higher and, therefore, best utilize the effects of ICK in addition to DCK. The dose covering the target was taken for the original SIMT‐VMAT plans and all the corresponding simulated VMAT plans. The original SIMT‐VMAT plans were found to fully covered the target by an average of 19.2 ± 0.3 Gy. As expected, the corresponding simulated plans with an induced setup errors of 0.5 mm/0.5°, 1 mm/1°, and 2 mm/2° were found to have the corresponding lower doses by 17.8 ± 0.8 Gy, 15.9 ± 0.9 Gy, and 12.6 ± 1.5 Gy, respectively. These data were evaluated with a threshold of ICK by 15 Gy or higher in Figure 2. To fully utilize indirect cell killing methods, patient setup errors of at least 1.3 mm/1.3° in all 6 DOF must be maintained as shown by the background change of blue to red. Above this threshold of 1.3 mm/1.3° in all 6 DOF, ICK could potentially contribute to the tumor cells death.

**FIGURE 3:**
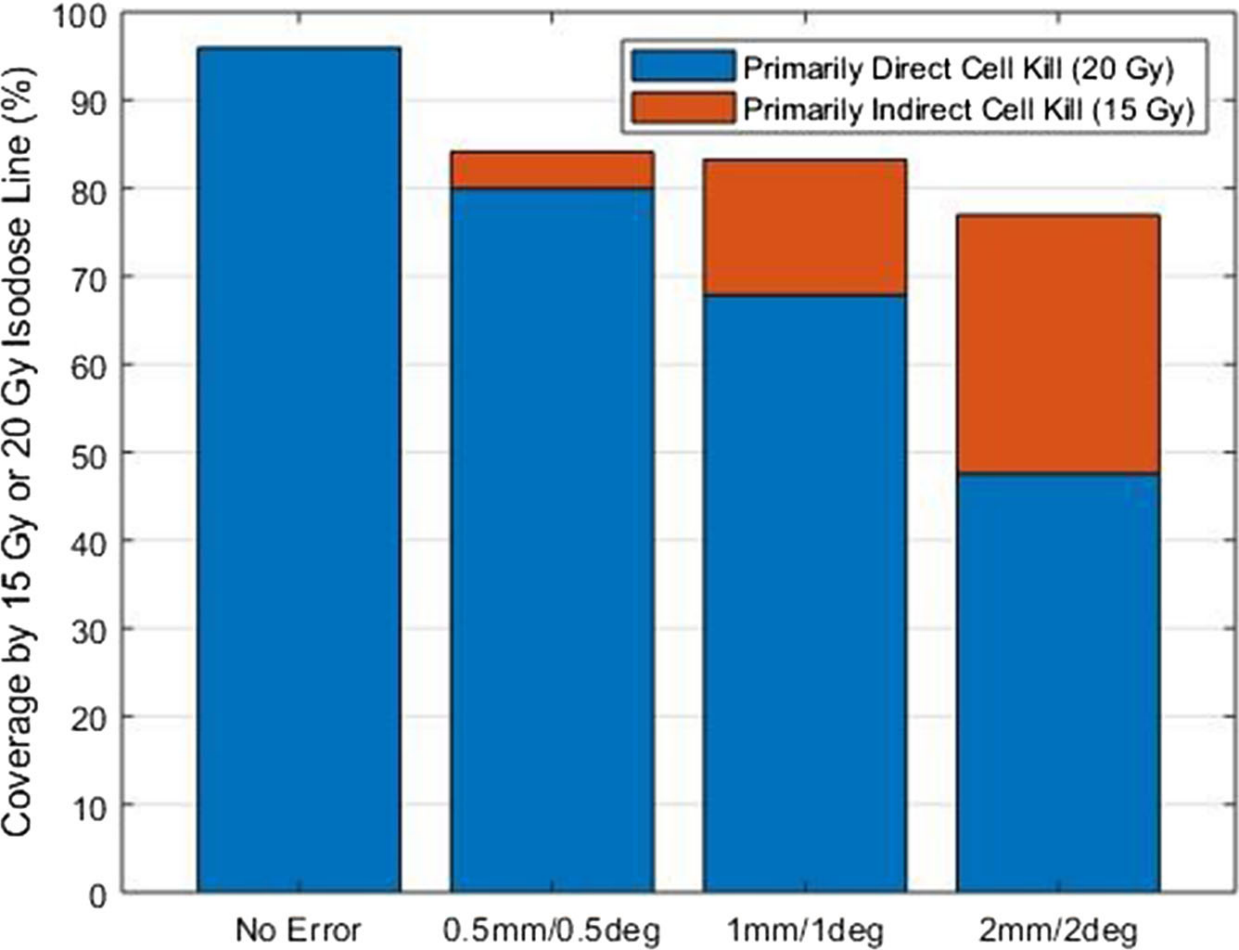
Illustration of the target coverage by the 15 and 20 Gy isodose lines. The blue represents the coverage obtained by 20 Gy isodose line, which is assumed to be primarily responsible for DCK. The red represents the 15 Gy isodose line or more, which is assumed to be primarily inducing ICK in addition to DCK. Without considering setup uncertainty, the target is nearly fully covered by the prescription isodose line (20 Gy) and could receive full effects of DCK. With induced setup errors, the coverage of the target by the prescription decreases, but is somewhat counterbalanced by the effects of ICK by 15 Gy or higher

The contributions of the DCK versus ICK methods are explained in Figure 3. The Boolean structure of the 20 Gy IDV and PTV is considered primarily DCK contributions, whereas the Boolean structure of the 15 Gy IDV and PTV minus the 20 Gy IDV is considered contributions from primarily ICK. For the original plans, the PTV was covered almost completely by the 20 Gy IDV for 97.97 ± 3.52% and no coverage by the 15 Gy IDV. For the corresponding simulated VMAT plans of 0.5 mm/0.5°, 1 mm/1°, and 2 mm/2° of setup errors, the 20 Gy IDV coverage was 80.0 ± 28.5%, 67.9 ± 21.6%, and 47.6 ± 23.6% and the 15 Gy IDV coverage was 4.2 ± 13.1%, 15.4 ± 10.8%, and 29.4 ± 14.6%, respectively. The contribution of DCK decreases as that of ICK increases, with 2 mm/2° having the worst overall target coverage, but most importantly, adding some ICK contributions. The DCK is somehow compensating for much of the dosimetric discrepancy up to 1.0 mm/1.0°. There is acceptable target coverage (>15 Gy), providing a better combined coverage, and therefore, potentially providing positive outcomes.

**FIGURE 4:**
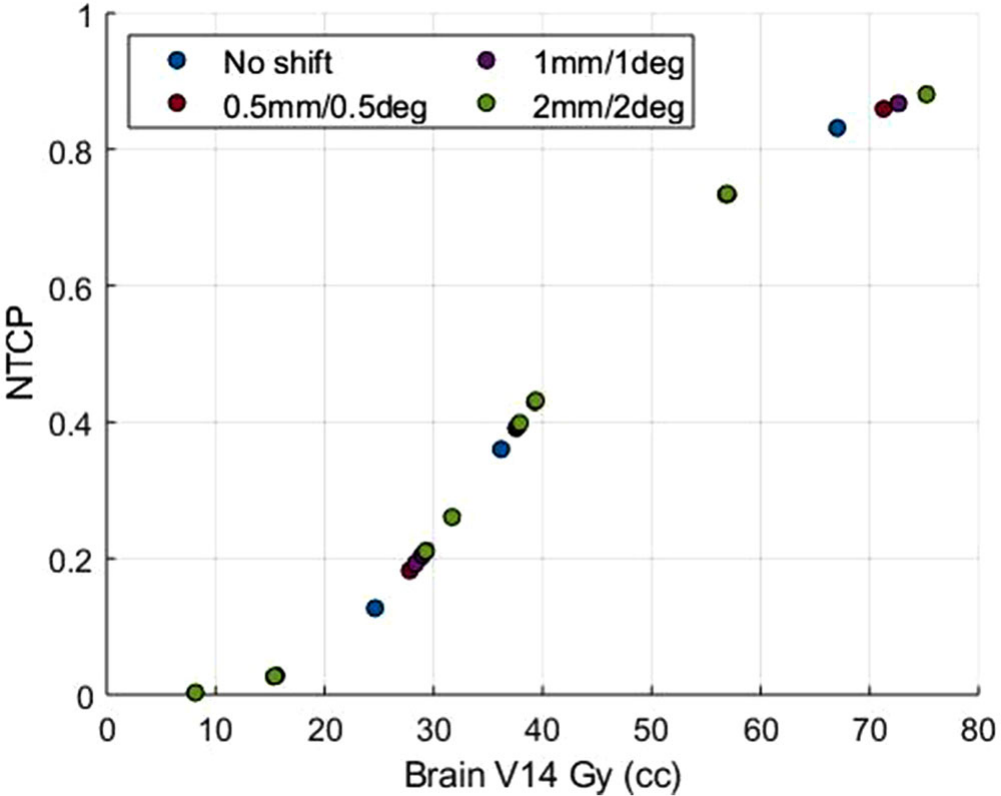
Comparison of NTCP of whole‐brain V14Gy for original SIMT‐VMAT plans and the corresponding simulated VMAT plans with induced setup uncertainties of 0.5 mm/0.5°, 1 mm/1°, and 2 mm/2° in all 6 DOF. Values of Vx50 and γ50 were obtained from literature as 45.8 cc and 0.88, respectively.^37^ The blue points represent NTCP with no setup uncertainty, the red ± 0.5 mm/0.5°, the purple ±1 mm/1°, and the green ± 2 mm/2° as a function whole‐brain V14Gy. There is no clinically significant increase in NTCP due to patient setup errors, however, NTCP of normal brain increases significantly as a function of V14Gy for those patients with increasing V14Gy above 30 cc

It has been observed that patient misalignment errors have minimal or no effect on NTCP of brain as shown in Figure 4. For each of the patients, the NTCP was calculated and compared with the whole brain receiving V14 Gy. For a majority of patients, there is not much of an increase in values of NTCP with any setup uncertainties up to 2 mm and 2° in each direction. For instance, the increase of NTCP of normal brain toxicity at setup errors of 2 mm and 2°in each direction had an absolute difference of less than 0.4% compared to the original plan with no setup errors, suggesting minimal brain toxicity risk while still resulting in clinical local tumor control. Based on percent differences in NTCP, it was determined that brain toxicity was 1.3, 1.5, and 1.7% more likely for 0.5 mm/0.5°, 1 mm/1°, and 2 mm/2° simulated plans. However, it is apparent that NTCP does increase with whole‐brain V14Gy, but the increase due to setup errors is not clinically significant for lower brain V14 Gy cases (see Figure 4).

The dose to OAR fluctuated depending on the distribution and orientation of the lesions to the immediately adjacent organs. Many cases resulted in substantial increases in dose to OAR with increased dose to hippocampi, brainstem, and optic apparatus up to 3, 2, and 1 Gy, respectively, due to patient setup errors using SIMT‐VMAT.

## 4 DISCUSSION

In addition to DCK, the effects of ICK responsible for providing better local tumor control rates for SIMT‐VMAT plan are explored with consideration of dosimetric discrepancies due to patient setup errors. This model may guide the determination of the setup uncertainty limits for the treating physicians that could utilize ICK effect to maintain acceptable tumor coverage in the SIMT setting. This was done by determining the dose levels that fully cover the target for SIMT‐VMAT plans with no setup uncertainty compared to clinically observable patient setup errors of 0.5 mm/0.5°, 1 mm/1°, and 2 mm/2° in all 6 DOF. Setup limits of at least 1.3 mm and 1.3° or better in all 6 DOF was found as the threshold to maintain an acceptable target dose while including the effects of ICK. The amount of contribution of both direct and ICK was also modeled using Boolean structures, so that 20 Gy IDV was assumed to be primarily responsible for DCK, while the 15 to 20 Gy IDV was assumed to be primarily contributing due to ICK. As setup uncertainty increases, the contribution of ICK increases (up to 15 Gy IDV coverage) and, therefore, tumor cell death by devascularization. The apparent dosimetric disparity from losing target coverage of the prescription dose is partially mitigated by incorporating the concept of secondary cell kill. In addition, the effects of setup uncertainty on the normal brain were modeled using NTCP. No clinically significant increase of NTCP of the brain due to setup errors was observed, while clinically significant increases in OAR are possible for these setup uncertainties due to the proximity of the organs, therefore, the planner should pay attention to those OAR.

Treating m‐bm with a single‐isocenter plan comes with many challenges, including dosimetric disparities, due to patient positioning errors.^14^, ^15^, ^16^, ^17^, ^18^, ^19^, ^20^ This presents concerns in terms of local tumor control and unexpected dosing to the normal brain and other adjacent critical structures in the brain as described above. The QUANTEC guidelines for normal brain tissue cite a study relating V12Gy to radiation‐induced necrosis, where the risk of NTCP increases from 23% for V12Gy between 0 and 5 cc and 54% for V12Gy at 10 to 15 cc.^39^, ^40^ It should be noted that dose to whole brain to a certain dose level is primarily on treated volume rather than number, shape, or location of lesions.^41^ Several recent clinical outcome studies have reported positive results of higher tumor local control rates of SIMT‐VMAT treatment that do not align with the presence of these dosimetric disparities.^21^, ^22^, ^23^, ^24^, ^25^ For instance Alongi et al. used Varian single‐isocenter VMAT in the treatment of 43 patients with m‐bm and performed a clinical follow‐up study within 6 months. They observed that 60% of the patients with partial or complete responses and 40% with stable disease control, though the medial overall survival had not yet been reported.^22^ Other studies have found similar tumor local control rates for linac‐based brain SRS using single‐isocenter plans.^21^, ^23^, ^24^ These clinical observations lead to the consideration of how the radiobiology of single fraction, high‐dose SRS could play an important role in SIMT‐VMAT for treating m‐bm.

Recently, Sperduto et al. discussed the high control rates of stereotactic body radiation therapy (SBRT) and SRS and suggested the concept of ICK.^32^ They discuss the roles biological models play in the evaluation of outcomes in SRS treatments. Their results suggest that a single dose of 15 Gy or higher correlates with indirect death of hypoxic cells by modes of devascularization and potentially radiation‐induced immune enhancement. The authors conclude that in addition to DCK, the secondary cell death by modes of devascularization may be the mechanism of interest that providing success for SRS/SBRT. Other studies concluding devascularization as a relevant form of secondary cell death have dated back to the 1950s.^42^, ^43^, ^44^ Other means of secondary cell death must also be considered in addition to devascularization. These treatments have shown to promote antitumor immunity with the expression of immune modulation molecules such as immunostimulatory cytokines.^45^, ^46^ However, literature states that for brain metastases treated with SRS alone, antitumor immunity was not sufficiently enough to strongly correlate to the outcomes of the treatment.^47^ In addition, radiation‐induced tumor‐specific immunity does not present until 1–2 weeks after high doses of radiation.^48^ The literature cited in this article supports secondary cell death occurring 2 to 3 days after 20 Gy irradiation, suggesting that the ICK via devascularization could contribute predominantly.^28^ This literature also cited the abscopal/bystander affects as an alternative mode of secondary cell death. Though, it is likely that ICK could be a combination of all the mentioned mechanisms above. This must be considered when evaluating dosimetric uncertainties due to setup errors for SIMT‐VMAT treatments. Both direct and ICK could be playing roles in tumor cells death, resulting in the higher local control rates reported by the mentioned clinical observations. This simulation study demonstrated that an acceptable target dose could be maintained when small setup uncertainties exist because the target coverage by a dose of 15 Gy or higher is still maintained and therefore cell death by devascularization. It is, therefore, suggested that if setup errors cannot be maintained between 1.3 mm/1.3° in all 6 DOF, alternative treatment methods to m‐bm should be used. It is recommended to use either a dual‐isocenter approach or traditional individual isocenter to each tumor methods instead.^49^, ^50^

Though this study brings perspective to radiobiological effects that exist when treating m‐bm via SIMT‐VMAT plan, some limitations must be considered. Uncertainty evaluated in this report was only due to patient setup uncertainty due to using a single‐isocenter plan. Other uncertainties are present such as target delineation errors and partial volume effects for the small lesions. This could also contribute to the apparent loss of target(s) coverage by the prescription isodose line and merits future investigation. Though positive clinical outcomes were evident, there were still inconsistencies between the literature for reported local control rates. This study is describing single fraction treatments resume, while the literature supporting positive local control range from single to five fractions with a variable number of patients receiving WBRT or surgical resection in addition to SRS treatments. They also use larger margins of 2–3 mm around the GTV, which could be accounting for some levels of setup uncertainty, though the effect on the normal brain is not mentioned.^21^, ^22^ These high local control rates are still useful in describing the ICK effects that could be taking place with high dose per fraction treatments, even with patient setup errors were evident, although cannot be predictive for all patient cohorts. As mentioned previously, an assumption is made in this study that the 15 Gy or higher IDV is a parameter of choice to describe the effect of ICK. However, there are some studies suggesting a single dose of 10–12 Gy as a threshold for ICK.^28^, ^29^, ^30^ Moreover, it is actually a combination of both ICK and DCK methods that take place between 15 and 20 Gy, though for simplification, just ICK is considered. Even though, the cut‐off value of 15 Gy was approximated for ICK, it would depend on tumor size, tumor type, and its radiosensitivity as well as immunologic effects. This is a major assumption that must be considered. This study is also limited by the TPS resolution limits when considering the tumor size of this patient cohort. These in combination will cause rounding of errors on IDV and Boolean structures, meaning some results for very small tumors will not be as accurate as those of larger tumors. Lastly, the LQ model is not an adequate representation of a dose‐response relationship for single high dose fraction SRS treatments, and though work has been done to create a relevant model, there is not currently a definite solution.^51^ Though 15 Gy is a potential parameter to consider, it may be difficult to directly apply this value all the time. The value is approximate and other parameters, such as alternative modes of secondary cell kill, could cause deviations in this value. There is a room to further investigate this in the future. The studies mentioned were done with human fibrosarcoma xenografts that grew in the legs of mice up to 6–7 mm in diameter and irradiated with single fractions, where some brain mets are larger in size.^25^, ^26^, ^27^, ^28^, ^29^, ^30^, ^31^ Therefore, it must be recognized that this is a simulation study, therefore, the results reported are not predictive of current patient treatment. Further clinical studies are warranted.

However, future research includes incorporating this ICK approach for SIMT‐VMAT plans in the treatment of m‐bm for reporting and clinical follow‐up of the patient's tumor local control and treatment‐related toxicity. It is also important to further investigate the radiobiological models of single‐fraction SRS treatments in terms of predicting tumor control probability (TCP) and how residual patient setup errors could affect the predicted treatment outcomes. Efforts have been made to model the TCP for single fraction treatments, but many still present with problems associated with unreliability of the current LQ model for single high dose of SRS that was historically generated for fractionated radiotherapy.^52^ Therefore, TCP depends on clinical observations rather than predicting local control rates. Tumor control can be further modeled with the relation of initial clonogenic cells and survival with patient setup uncertainty and secondary cell death, in addition to the direct cell death. It will also be useful to use cellular modeling to further understand the magnitude of the damage made by DCK versus ICK after single high dose of SRS treatment with respect to reduced tumor cell kill for some given dose levels as seen by tumor recurrences.

## 5 CONCLUSIONS

SRS treatment of m‐bm using a SIMT‐VMAT plan will result in dosimetric discrepancies due to immitigable residual patient positioning uncertainties. In addition to DCK, the ICK due to the vascular damage and/or enhanced intratumor immune response with a single high‐dose of 15 Gy or higher tumor coverage could potentially compensate for patient setup errors up to ±1.3 mm/1.3° in all six directions. It could still present positive clinical outcomes for the tumor local control along with no clinically significant increases in normal brain toxicity. Clinical follow‐up results of the m‐bm patients treated via a SIMT‐VMAT SRS plan as a function of residual patient setup errors that incorporate ICK in addition to DCK is warranted.

## AUTHOR'S CONTRIBUTIONS

AP and DP conceived the project and generated treatment plans. AP simulated uncertainties, performed modeling, collected, and analyzed the data. DP, DF, WSC, and MR provided clinical expertise and supervision of the article. AP and DP drafted the manuscript and all coauthors revised and approved the final manuscript.

## CONFLICT OF INTEREST

The authors declare no conflict of interest.

## Supporting information

SUPPORTING INFORMATION

## Data Availability

Research data are not shared.
