# Supplementary material for: Predicting the effect of indirect cell kill in the treatment of multiple brain metastases via single‐isocenter/multitarget volumetric modulated arc therapy stereotactic radiosurgery
Source: J Appl Clin Med Phys. 2021 Sep 8;22(10):94–103. doi: 10.1002/acm2.13400 (PMC8504608; doi:10.1002/acm2.13400)
Supplement: Supplementary file 1 — SUPPORTING INFORMATION [file ACM2-22-94-s001.docx]

**Predicting the Effect of Indirect Cell Kill in the Treatment of Multiple Brain Metastases via Single-Isocenter/Multi-Target Volumetric Modulated Arc Therapy Stereotactic Radiosurgery**

Allison N Palmiero, MS, Denise Fabian, MD, Marcus E Randall, MD, William St Clair, MD, PhD, and Damodar Pokhrel, PhD

Medical Physics Graduate Program, Department of Radiation Medicine

University of Kentucky, Lexington KY

**Corresponding Author**:

Damodar Pokhrel, PhD, DABR

Associate Professor of Medical Physics

Medical Physics Graduate Program

Department of Radiation Oncology

University of Kentucky, Lexington KY

Phone no: (859) 323-7599

Email: [damodar.pokhrel@uky.edu](mailto:damodar.pokhrel@uky.edu)

**Funding:** None

**Key words:** SIMT-VMAT SRS, multiple brain metastases, set up uncertainties, direct/indirect cell kill

**Short title**: Indirect Cell Kill in Radiosurgery of Multiple Brain Metastases via SIMT-VMAT

**Author’s Contributions:** AP and DP conceived the project and generated treatment plans. AP simulated uncertainties, performed modeling, collected and analyzed the data. DP, DF, WSC and MR provided clinical expertise and supervision of the paper. AP and DP drafted the manuscript and all co-authors revised and approved the final manuscript.
